# Study on Dynamic Fermentation of Oat Silage Assisted by Exogenous Fibrolytic Enzymes

**DOI:** 10.3390/plants13010006

**Published:** 2023-12-19

**Authors:** Wei Liu, Shuai Du, Lin Sun, Zhijun Wang, Gentu Ge, Yushan Jia

**Affiliations:** 1 Key Laboratory of Forage Cultivation, Processing and High Efficient Utilization of Ministry of Agriculture and Rural Affairs, Inner Mongolia Agricultural University, Hohhot 010019, China; liuwei0416996@126.com (W.L.); dushuai_nm@sina.com (S.D.); zhijunwang321@126.com (Z.W.); gegentu@163.com (G.G.); 2 Key Laboratory of Grassland Resources, Ministry of Education, Inner Mongolia Agricultural University, Hohhot 010019, China; 3 Department of Grass Science, Inner Mongolia Agricultural University, College of Grassland, Resources and Environment, South Campus, Hohhot 010019, China; 4 Inner Mongolia Academy of Agricultural and Animal Husbandry Sciences, Hohhot 010031, China; sunlin2013@126.com

**Keywords:** oat, silage, xylanase, cellulase, bacteria community

## Abstract

Based on the low content of water-soluble carbohydrate (WSC) and lactic acid bacteria (LAB) attachment in oat raw materials, we assumed that the neutral detergent fiber (NDF) content of oat can be reduced by adding cellulase or xylanase. The concentration of metabolizable sugars will be increased, which will assist the oat’s bacterial community in fermentation and obtain a better quality of oat silage. After wilting the oat, it was treated as follows: (1) distributed water (CK); (2) silages inoculated with xylanase (X); and (3) silages inoculated with cellulase (C), ensiling for 3, 7, 14, 30, and 60 days. Cellulase and xylanase treatments both alter the fermentation and nutritional quality of ensiled oat, resulting in lower NDF, acid detergent fiber (ADF), cellulose, and hemicellulose contents, increased lactic acid and acetic acid contents, and a significant decrease in ensiling environment pH. The bacterial community undergoes significant changes with cellulase and xylanase treatments, with a significant increase in *Lactobacillus* abundance in the C_14, X_30, C_30, X_60, and C_60 treatment groups, while *Weissella* abundance gradually decreases with longer ensiling times. Two exogenous fibrolytic enzymes also alter the bacterial diversity of ensiled oat, with different bacterial species and abundances observed in different treatment groups. Ensiled oat treated with cellulase and xylanase experiences significant changes in its own bacterial community, particularly in the abundance of *Lactobacillus*. These changes result in improved fermentation and nutritional quality of oat, but the higher metabolism levels observed after 60 days of ensiling with cellulase treatment may lead to energy loss.

## 1. Introduction

Plants are trophosomes formed through photosynthesis, which is rich in various nutrients and enables various microorganisms to survive. At the same time, the activities of many microorganisms also promote plant growth. These microorganisms related to plants are often defined as pathogens, mutualists, or commensals [1]. The plant microbiota mainly covers three parts: the rhizosphere, the internal tissue of the plant body, and the plant surface (stem and leaves) [2]. Silage was a feed product that used the aboveground parts of plants to improve the acidity of the environment through LAB fermentation to achieve long-term preservation [3]. The microbial attachment content and types of plants are important factors for the safe fermentation of plants [4], so it is necessary to discuss the changes in the microbial community of plants during the silage fermentation process. However, many silage materials are not suitable for silage fermentation due to their microbiota or the number of LABs. It is a good choice to use external additives to improve fermentation, such as LAB [5], *Lactobacillus plantarum* [6], organic acid salts [7], exogenous fibrolytic enzymes [8], etc. The microbial community attached to plants is an important basis for silage fermentation and plays a crucial role in the silage process. Many studies have found that exogenous microbial communities can change the microbial community of silage, but often overlooked is the discussion of the microbial community attached to plants [9]. This may be a crucial part, as its synergistic, antagonistic, or symbiotic effects with exogenous microbial communities can affect the fermentation process of silage. Not only microorganisms, but silage fermentation is influenced by many factors, such as water content, sugar content, temperature, etc. However, the carbon source is one of the most important factors in microbial fermentation, as the vast majority of microorganisms cannot grow without it [10]. In addition, the WSC gradually decreased due to microbial activity during the ensiling process, and about 70 g/kg of WSC was lost compared to hay samples [11].

Oat (*Avena sativa* L.) is a common cereal and forage crop, and it represents a significant feed source on a global scale. However, due to the different cultivation sites or harvest stages of oat, most of them had lower WSC and lactic acid bacteria, which were not unsuited to silage fermentation. But oat, as one kind of Poaceae plant, was rich in cellulose and hemicellulose [11], which provided sufficient possibility for fermentation substrate. Cellulases (endo-1, 4-β-D-glucanases), produced by fungi, bacteria, and protozoans, are a kind of glycoside hydrolase that can hydrolyze the cellulose of plant cell walls. Products catalyzed by cellulase, such as sugars such as monosaccharides or oligosaccharides, can serve as direct sources of nutrition for bacteria. [12]. Xylanases (endo-1, 4-β-xylanase) have the ability to facilitate the breakdown of xylan into xylose through hydrolysis [13] and can also be directly utilized by microorganisms. Homofermentative lactic acid bacteria generally use glucose for fermentation, while heterofermentative lactic bacteria generally use pentose for fermentation [14]. Therefore, treatment with cellulase and xylanase may increase the concentration of metabolizable sugars in silage to assist LAB fermentation.

Recent studies have shown that cellulase can improve the fermentation quality of hybrid Pennisetum silage feed. When compared with the utilization of *Lactobacillus plantarum* and sucrose treatment, cellulase demonstrates superior nutritional preservation and an enhanced capacity to inhibit protein hydrolysis [15]. Cellulase treatment hydrolyzes cellulose into glucose, and the LAB can directly utilize it [15]. Compared with the no-additives treatment, the cellulose treatment increased lactate content, decreased pH, and significantly increased the abundance of *Lactobacilli* [16]. Xylanase converts the xylan in the cell wall into xylose and provides a microbial fermentation substrate. The application of xylanase as a pre-treatment method for agricultural silage enhances its nutritional value by decreasing the concentration of hemicellulose in the cell wall [17]. Xylanase treatment of silage promotes the production of xylose, which is then fermented by *Lactobacillus* into lactic acid to facilitate the preservation of the silage [18]. Based on the low WSC and LAB content of oat, which may not be conducive to silage fermentation, this study will explore whether adding cellulase and xylanase to oat silage can decompose cellulose or hemicellulose to provide sufficient substrate for LAB to ferment, promote microbial growth, and improve fermentation quality.

## 2. Results

### 2.1. Microbial Community Attached to Fresh Oat

**Figure 1:**
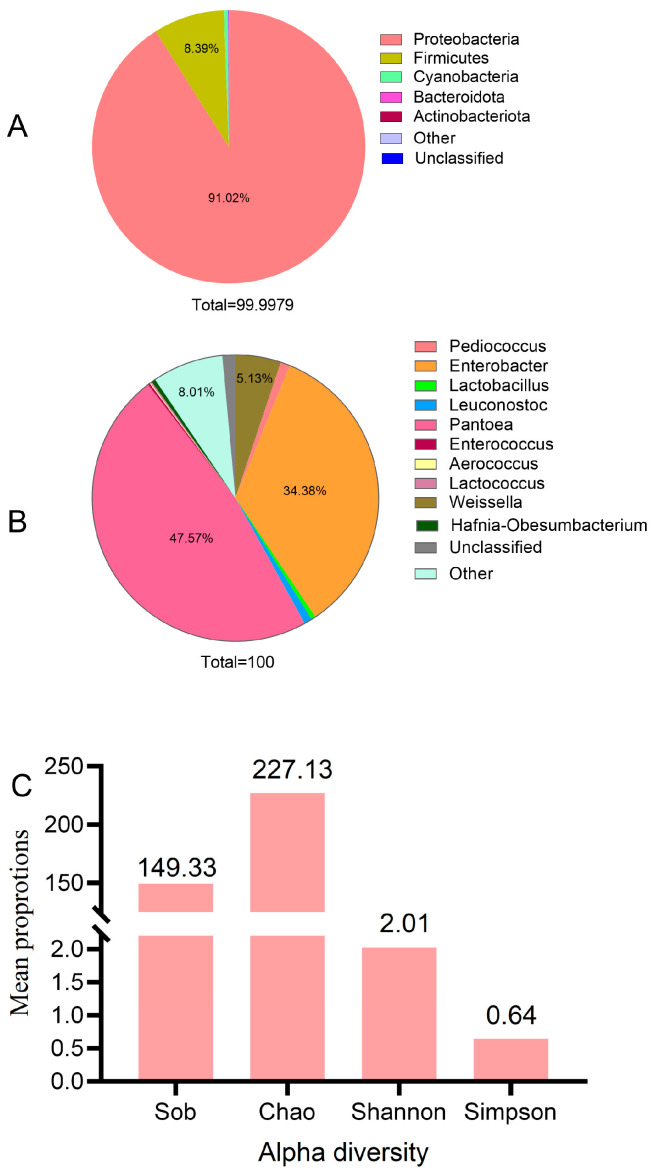
The pie chart of fresh oat’s microbial community (**A**,**B**) expressed abundance at the phylum level and genus level, respectively, and (**C**) expressed alpha diversity of fresh oat.

The abundance and alpha-diversity of the bacterial community carried by fresh oats are shown in Figure 1. The main bacterial community of fresh oat was Proteobacteria, with an abundance of 91.02% at the phylum level, followed by Firmicutes, with an abundance of 8.39%. On the genus level, the bacterial community of fresh oat was mainly *Pantoea*, *Enterobacter*, and *Weissella*, with an abundance of 47.57%, 34.38%, and 5.13%, respectively. The fresh oat Sob, Chao, Shannon, and Simpson indexes were 149.33, 227.13, 2.01, and 0.64, respectively.

### 2.2. The Oat Silage of Quality and Microbial Count on Different Additives and Silage Days

**Figure 2:**
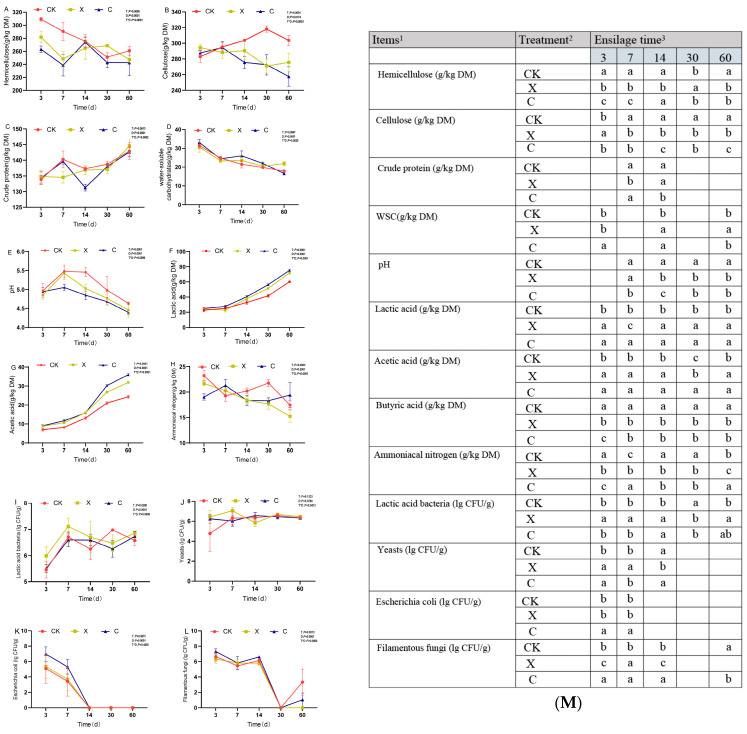
Dynamic changes in oat silage quality and microbial count with different treatment groups (**A**–**L**), (**A**): hemicellulose, (**B**): cellulose, (**C**): crude protein, (**D**): water-soluble carbohydrates, (**E**): pH, (**F**): lactic acid, (**G**): acetic acid, (**H**): ammoniacal nitrogen, (**I**): lactic acid bacteria, (**J**): yeasts, (**K**): *Escherichia coli*, (**L**): filamentous fungi. (**M**) was a table to illustrate the significance of the results, ^1^; WSC: water-soluble carbohydrate, ^2^; CK, no additive; X, inoculated with xylanase; C, inoculated with cellulase, ^3^; 3, 7, 14, 30, and 60 expressed different ensilage days, different lowercase letters indicate significant differences with different treatments.

Different silage exogenous fibrolytic enzymes and silage time had a significant impact on the quality and microbial count of oat silage. Compared with the control treatment, the DM content was significantly reduced by the treatment with xylanase (*p* < 0.05). Cellulase and xylanase treatments both reduced the NDF content of oat silage, and this declining trend was more obvious with the extension of silage time (Supplementary Material Table S2). After 60 days of silage and cellulase treatment, the NDF content was the lowest (551 g/kg DM) (Supplementary Material Table S2), significantly lower than the control treatment (*p* < 0.05). The ADF content was significantly lower than the control treatment after 14, 30, and 60 days of silage under additive treatment (*p* < 0.05), especially after 60 days of silage, when the ADF content reached the lowest level of 308 g/kg DM under cellulase treatment (Supplementary Materials). Silage time had no significant effect on the ADL of oat silage. After 60 days of silage (Supplementary Material Table S2), the ADL content of the xylanase treatment was significantly lower than that of the control treatment (*p* < 0.05). Cellulose and hemicellulose showed a downward trend with increased silage time, except for the control treatment (Figure 2A,B). Compared with the control, different exogenous fibrolytic enzyme treatments all reduced the cellulose and hemicellulose contents in oat silage. The results showed that the additives, silage days, and their interaction significantly changed the cellulose and hemicellulose contents in oat silage. After 60 days of silage, the cellulose content reached a minimum with cellulase treatment at 258 g/kg DM. The additive treatment did not have a significant effect on the CP content of oat silage, but with the extension of silage days, the CP content showed an upward trend, especially after 60 days of silage, when the CP content increased (Figure 2C). The WSC content decreased significantly with the extension of silage time (Figure 2D). After 60 days of silage, the WSC content (22 g/kg DM) under xylanase treatment was significantly higher than the control, but the WSC content under cellulase treatment did not increase. Supplementary Materials for more data reference (DM, NDF, ADF, ADL).

With the increase in silage time, the pH of silage increased first and then decreased (Figure 2E). The pH of silage was significantly reduced by cellulase and xylanase at 14, 30, and 60 days of silage. The C_60 group treatment has reached a minimum of 4.39. Compared with the control group, both xylanase and cellulase treatments significantly increased the contents of lactic acid and acetic acid in silage (Figure 2F,G). Additives, silage days, and their interactions had significant effects on the contents of lactic acid and acetic acid in oat silage. The ammonia nitrogen content in silage was affected by additive treatment and silage days. With the increase in silage time, the ammonia nitrogen content showed a decreasing trend (Figure 2H), and the lowest histamine nitrogen content in the X_60 treatment was 15 g/kg. The contents of LAB in silage tended to rise with the increase in silage time (Figure 2I). Similarly, exogenous fibrolytic enzymes also had a significant impact on the contents of LAB. The contents of LAB in the X_60 and C_60 treatment groups were lower. The changing trend of yeast content in oat silage was relatively smooth under the treatment of additives and silage days, and the single factor treatment had no significant effect on yeast (Figure 2J). The content of *Escherichia coli* in oat silage decreased significantly with the increase in silage time (Figure 2K), and *Escherichia coli* disappeared after 14 days of silage. The exogenous fibrolytic enzyme treatment had no significant effect on the content of *Escherichia coli* in oat silage. The contents of Filamentous fungi in ensilage decreased with the increase in ensilage time and disappeared after 30 days, but Filamentous fungi appeared again in the CK_60 and C_60 treatment groups with low contents (Figure 2L).

### 2.3. Dynamic Changes of Microbial Community with Different Treatment Groups

**Figure 3:**
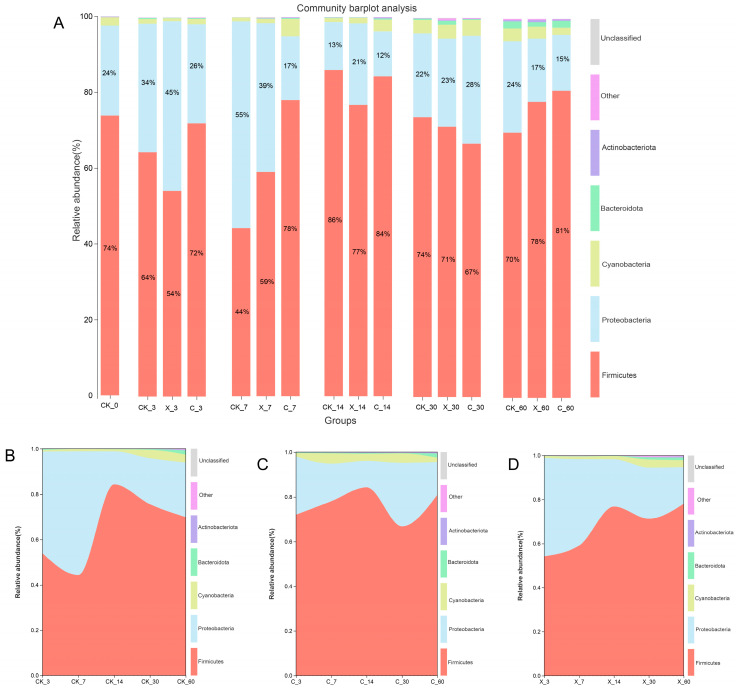
Bacterial community composition (**A**) and succession (**B**–**D**) at the phylum level in silage during the ensiling process. CK, no additive; X, inoculated with xylanase; (**C**), inoculated with cellulase; 3, 7, 14, 30, and 60 expressed different ensilage days; 0 expressed oat before ensiling; for example, CK-3 expressed that there was no additive after 3 days of silage.

**Figure 4:**
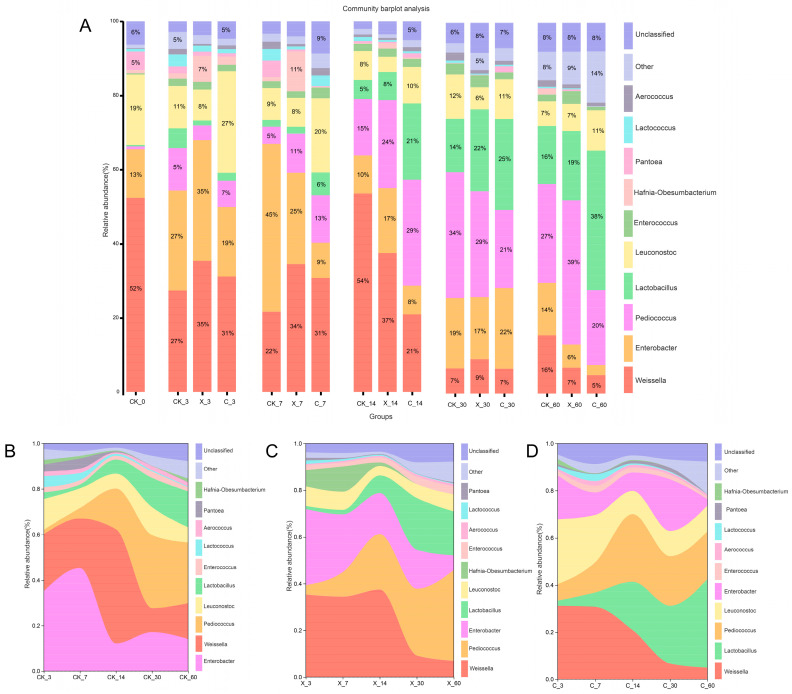
Bacterial community composition (**A**) and succession (**B**–**D**) at the genus level in silage during the ensiling process. CK, no additive; X, inoculated with xylanase; (**C**), inoculated with cellulase; 3, 7, 14, 30, and 60 expressed different ensilage days; 0 expressed oat before ensiling; for example, CK-3 expressed that there was no additive after 3 days of silage.

Different silage days and exogenous fibrolytic enzyme treatments all changed the bacterial abundance in oat silage, both at the phylum and genus levels. Exogenous fibrolytic enzymes have shown different effects on the abundance of the oat-silage microbiota at different stages. Compared with the control, X_7, C_7, C_14, X_60, and C_60 treatments increased the abundance of Firmicutes in silage and decreased the abundance of Proteobacteria (Figure 3A). But the X_3, X_14, X_30, and C_30 treatment groups reduced the abundance of Firmicutes in silage and increased the abundance of Proteobacteria. The abundance of cyanobacteria in silage is mainly distributed after 14, 30, and 60 days of silage. From the analysis of the river map, both cellulase and xylanase treatments showed varying degrees of differences compared to the control treatment under different silage days, but after 14 days of silage, the Firmicutes showed higher abundance (Figure 3B–D). Cellulase and xylanase had a significant impact on the abundance of bacterial communities in oat silage at the genus level. Compared with the control, X_14, C_14, X_60, and C_60 treatment groups significantly reduced the abundance of *Weissella* in oat silage and significantly increased the abundance of *Lactobacillus* in silage (Figure 4A). X_7, C_7, X_60, and the C_60 groups significantly reduced the abundance of *Enterococcus* in silage compared to the control treatment, but the C_30 treatment group did not reduce the abundance of *Enterococcus* in silage. The abundance of *Pediococcus* significantly increased in silage in the X_7, C_7, X_14, and C_14 treatment groups compared to the control treatment group, but the X_30 and C_30 treatment groups reduced the abundance of *Pediococcus*. From the analysis of the river map, *Weissella*, *Enterococcus*, and *Leuconostoc* showed a downward trend with the extension of silage time, while Lactobacillus and *Pediococcus* showed an upward trend with silage time, and the abundance of *Lactobacillus* significantly increased at cellulase treatment with the extension of silage time (Figure 4B–D).

**Figure 5:**
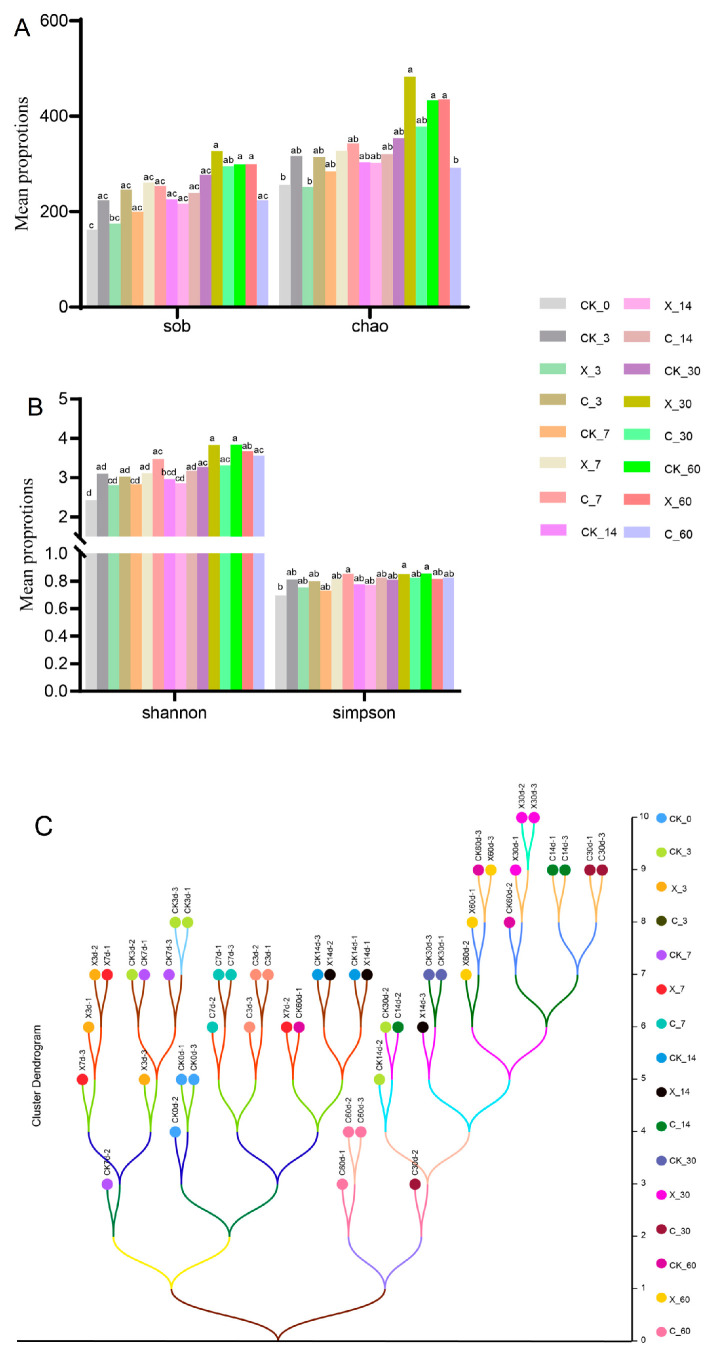
Differences in bacterial (**A**,**B**) community diversity and richness in oat silage with different additive treatments and ensilage days. (**A**) Sob and Chao index. (**B**) Shannon and Simpson index. The cluster dendrogram analysis of bacterial communities (**C**) on operational taxonomic units (OTUs) level in oat silage with different additive treatments and ensilage days. CK, no additive; X, inoculated with xylanase; C, inoculated with cellulase; 3, 7, 14, 30, and 60 expressed different ensilage days; 0 expressed oat before ensiling; for example, CK_3 expressed that there was no additive after 3 days of silage. Different lowercase letters indicate significant differences with different treatments.

The α-diversity of the bacterial community of oat silage is shown in Figure 5A,B. The Sob index of oat silage did not show significant effects under different additives (cellulase and xylanase) treatment. The Sob index of the X_30, CK_60, and C_60 treatment groups significantly increased compared to the CK_0 and X_3 treatment groups and fresh oat. Except for the C_60 treatment group, which significantly reduced the Chao index of oat silage, the addition of additives did not result in a noticeable effect on the Chao index of the silage. The Chao index of oat silage with the X_30, CK_60, and X_60 treatment groups was significantly higher than the CK_0, X_3, and C_60 treatment groups and fresh oat. During the same silage period, cellulase and xylanase treatments did not significantly affect the Shannon and Simpson index of oat silage. The Shannon index of silage in the X_30 and CK_60 treatment groups was significantly higher than the CK_0 and X_3. CK_7 and X_14 treatment groups and fresh oat, the Simpson index of silage with the C_7, X_30, and CK_60 treatment groups was significantly higher than the CK_0 group and fresh oat. Based on the presence and abundance of species, we conducted cluster dendrogram analysis on silage samples after different treatments (Figure 5C). There were two main branches here, one of which evolved after 3 days and 7 days of silage and the other after 14, 30, and 60 days of silage, indicating significant differences in the presence and abundance of species between these two branches. Both cellulase and xylanase treatments changed the clustering distance of oat silage, but it is more noteworthy that the C_60 treatment group has a far greater clustering distance compared to other treatments.

**Figure 6:**
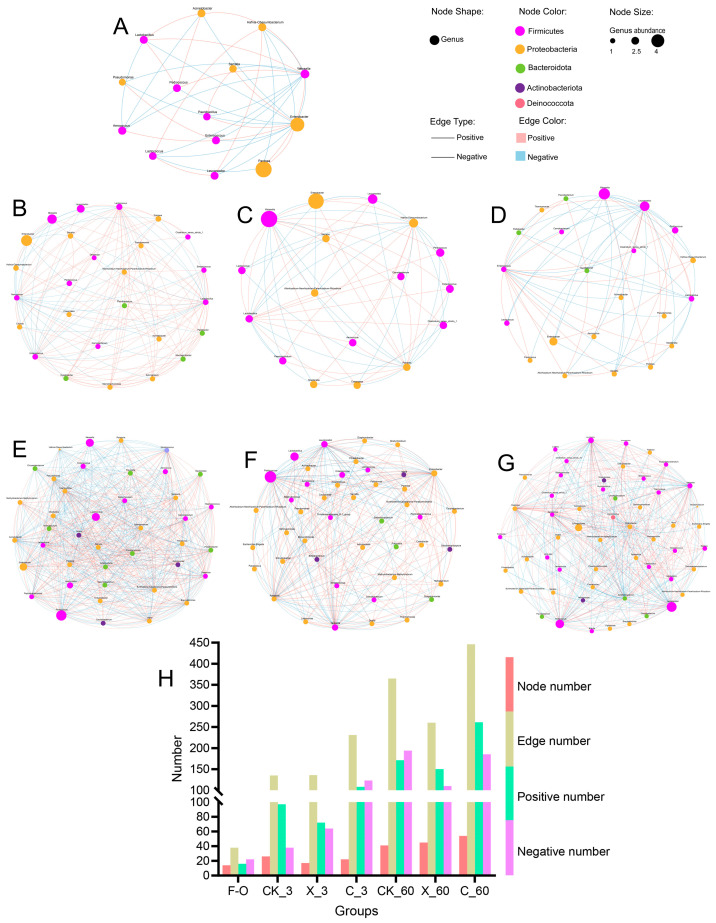
Cooccurrence patterns of the silage bacterial community during the ensiling process. The networks of result (**B**–**G**) were treated with different groups, which were CK_3, X_3, C_3, CK_60, X_60, and C_60 treatment groups, respectively. The different of node number, edge number, positive number, and negative number (**H**) were treated with different treatment groups. (**A**) were cooccurrence patterns of the fresh oat bacterial community. We selected bacterial communities with a genus level greater than 0.1% for mapping.

We compared the correlation network of oat silage after 3 days and 60 days; the CK_3 treatment group has higher connectivity and a positive correlation compared to the X_3 and C_3 treatment groups (Figure 6B–D); fresh oat was dominated by Proteobacteria mainly, showing lower connectivity and species (Figure 6A). Compared to 3 days of silage, 60 days of silage showed a higher species number and connectivity; the C_60 treatment group had the highest number of species and edge. No matter the fresh oat or silage oat, the Firmicutes and the Proteobacteria contributed mainly interrelationships.

**Figure 7:**
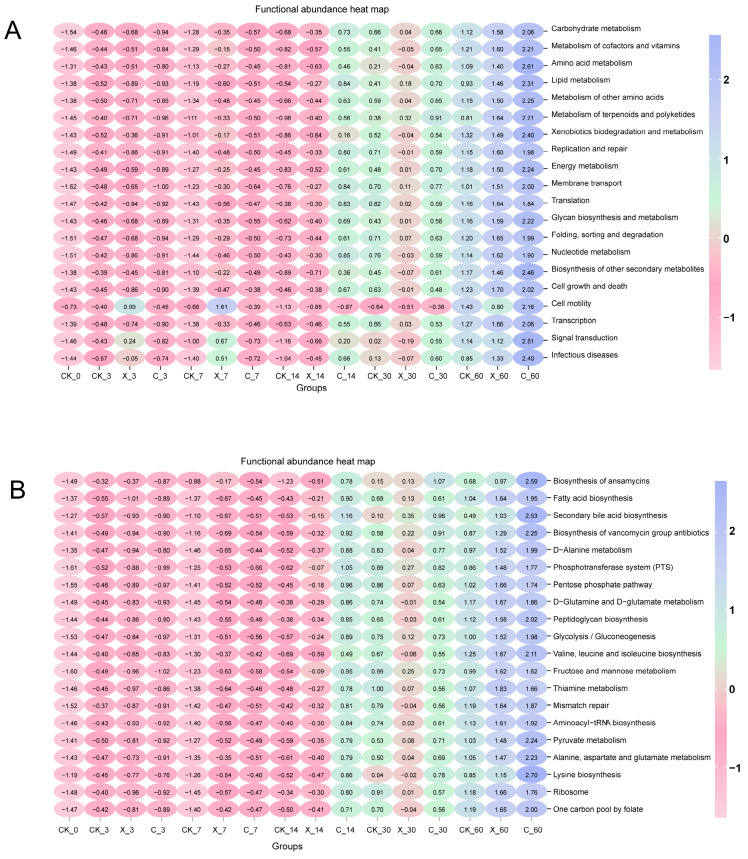
Functional abundance heat map on classification level_2 (**A**) and level_3 (**B**) in oat silage with different additive treatments and ensilage days. CK, no additive; X, inoculated with xylanase; C, inoculated with cellulase; 3, 7, 14, 30, and 60 expressed different ensilage days; 0 expressed oat before ensiling; for example, CK_3 expressed that there was no additive after 3 days of silage.

The functional abundance heat map is shown in Figure 7A,B. Similar metabolism was observed at both metabolic level 2 and metabolic level 3. The additive significantly changed the metabolic level of oat silage, especially the cellulase treatment, which significantly improved the various metabolic levels of silage. The CK_0 and CK_7 treatment groups had a very low metabolic level; the metabolic levels of various indicators in the C_60 treatment group were the highest, and with the extension of silage time, the metabolic levels of oat silage showed an upward trend.

**Figure 8:**
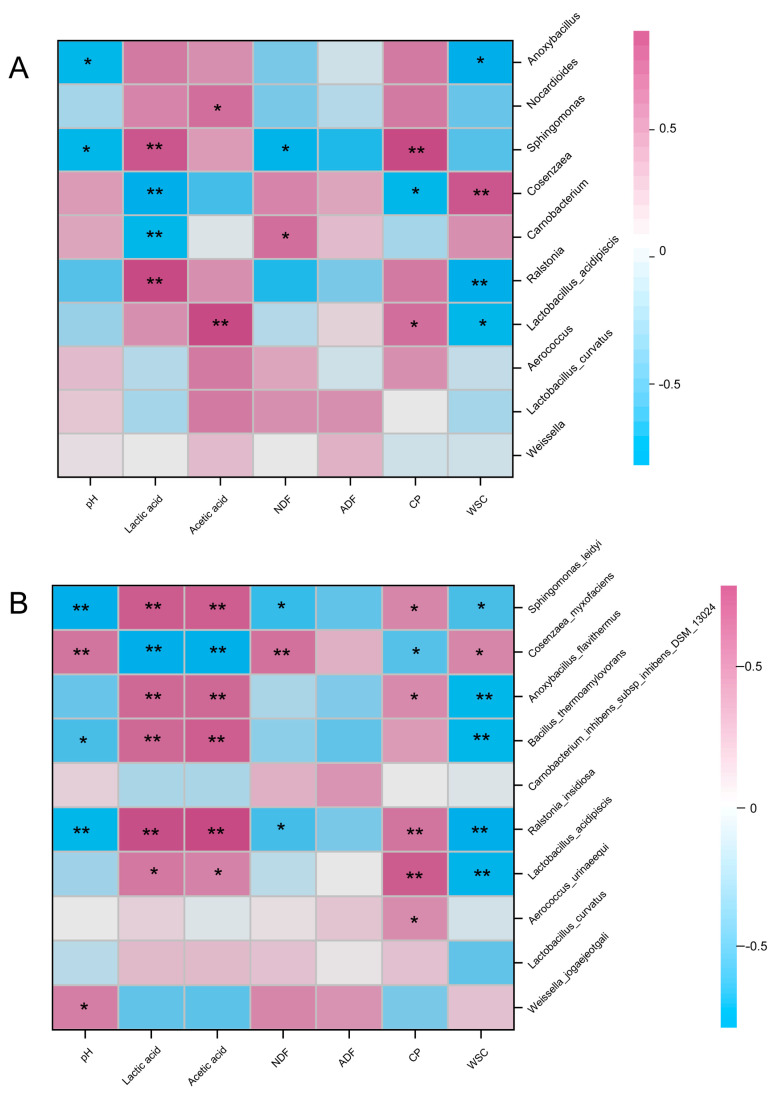
Spearman analysis between silage parameters and the top ten bacterial genus levels (**A**) and top ten species levels (**B**) with different additive treatments and ensilage days. NDF, neutral detergent fiber; ADF, acid detergent fiber; CP, crude protein; WSC, water-soluble carbohydrates. * *p* < 0.05; ** *p* < 0.01.

The spearman analysis shown in Figure 8 shows related relationships between silage parameters and bacterial genus or species under different additives and silage time treatments. Silage pH was positively correlated with the abundances of *Cosenzaea_myxofaciens* and *Weissella_jogaejeotgali* but negatively correlated with *Sphingomonas*, *Anoxybacillus*, *Ralstonia_insidiosa*, *Bacillus_thermoamylovorans*, and *Sphingomonas_leidyi* significantly (*p* < 0.05). The content of LA was positively correlated with the abundance of *Ralstonia*, *Sphingomonas*, *Lactobacillus_acidipiscis*, *Bacillus_thermoamylovorans*, *Ralstonia_insidiosa*, *Anoxybacillus_flavithermus*, and *Sphingomonas_leidyi*, notably, but negatively correlated with the abundance of *Cosenzaea*, *Carnobacterium*, and *Cosenzaea_myxofaciens*. The NDF content was strikingly positively correlated with the abundance of *Carnobacterium* and *Cosenzaea_myxofaciens* (*p* < 0.05) but negatively correlated with *Sphingomonas*, *Ralstonia_insidiosa* and *Sphingomonas_leidyi* (Figure 8). The CP content was strikingly positively correlated with the abundance of *Lactobacillus_acidipiscis*, *Sphingomonas*, *Aerococcus_urinaeequi*, *Ralstonia_insidiosa*, *Anoxybacillus_flavithermus*, *Cosenzaea_myxofaciens*, *Sphingomonas_leidyi* but negatively correlated with *Sphingomonas* and *Cosenzaea_myxofaciens*. The WSC content was positively correlated with the abundance of *Cosenzaea* and *Cosenzaea_myxofaciens* notably, but negatively correlated with *Bacillus_thermoamylovorans*, *Anoxybacillus_flavithermus*, and *Sphingomonas_leidyi* et al. (*p* < 0.05).

## 3. Discussion

### 3.1. Material Characteristics and Silage Quality

The range of raw materials for oat silage is close to other research results. Compared to previous studies, oat raw materials had a higher pH and a lower content of DM, WSC, NDF, and ADF (Supplementary Material Table S1), but the content of yeast and LAB was very low [19]. In comparison with this study, Li H.et al. [20] found that the CP content was higher, but Si W. et al. [19] found that the CP content was lower. The chemical composition of oat material was influenced by many factors, such as climate, soil, variety, harvest period, cultivation management [21], etc. The harvesting of oat materials during the heading period of this study potentially resulted in variations in nutritional composition. The presence of water-soluble carbohydrates (WSC) emerged as a critical factor influencing fermentation quality. The production of high-quality fermented silage feed necessitates an adequate WSC content (ranging from 60 to 80 g/kg DM) to facilitate the growth of lactic acid bacteria [22]. In this study, the WSC of oat silage feed was 62 g/kg (Supplementary Material Table S1), meeting the minimum requirement. However, during the silage process, if the fermentation substrate is insufficient, the WSC content will rapidly decrease, leading to fermentation failure and a decrease in nutritional quality [11]. Oat was rich in cellulose and hemicellulose (polysaccharides), which is beneficial for improving the fermentation quality of oat silage feed. The amount of LAB exceeding 5.0 log CFU/g during the silage process will achieve good preservation [23], but in this study, the LAB content was 1.51 log CFU/g FW (Supplementary Material Table S3), which was very poor for the fermentation of oat silage. Nonetheless, all treated oat silages exhibited favorable fermentation characteristics, with notable improvements observed in the cellulase and xylanase treatment groups. Consequently, the incorporation of exogenous fibrolytic enzymes effectively reduces fiber content, enhances metabolizable sugar levels, and ultimately promotes silage fermentation.

Nutritional quality is an important factor in evaluating the fermentation quality of silage, as the quality of silage nutrition directly affects the health and growth performance of animals. The DM content of oat silage was 290 g/kg FW, which is very close to the optimal dry matter content level for silage fermentation (300–400 g/kg FW) [24]. Cellulase and xylanase reduce the content of NDF and ADF in oat silage feed because they can hydrolyze plant cell walls to produce metabolizable sugars [23]. This is consistent with the research results of this article. Cellulase and xylanase both reduced the content of NDF and ADF in silage. After treatment with exogenous fibrotic enzymes, the cellulose and hemicellulose in silage showed a significant downward trend, which is consistent with previous research results [25]. The CP content in silage gradually increased with the extension of silage time, especially after 60 days of silage, which was different from previous studies [26]. This phenomenon may be due to fermentation, which can also convert some non-protein nitrogen into amino acids and amino compounds, resulting in a corresponding increase in CP content in silage feed [27]. The X_14, C_14, and C_60 treatment groups all increased the WSC content in oat silage, but there was no significant difference in other treatments. This may be due to the fact that the acidity in the environment during the oat silage process did not meet the standard values for inhibiting the activity of other harmful microorganisms [15]. In summary, cellulase and xylanase have played a positive role in improving the quality of oats.

The exogenous fibrotic enzyme treatment groups significantly reduced the pH of the silage and increased the content of lactic acid, acetic acid, and LAB. This may be due to the fact that both cellulase and xylanase treatments reduced the cellulose and hemicellulose content in the oat cell wall of the silage, providing sufficient fermentation substrates for LAB [28]. Different silage times did not affect the butyric acid content in silage, but exogenous fibrotic enzyme treatment reduced the generation of butyric acid content, which may be due to the inhibition of *Clostridium butyricum* growth by other dominant microorganisms with higher abundance [29]. The content of ammonia nitrogen in silage significantly decreased with the increase in silage time, indicating a transformation of this component under bacterial fermentation, which may also lead to an increase in CP content in silage [27]. *Escherichia coli* appeared in ensilage at 3 and 7 days and disappeared at 14, 30, and 60 days, possibly due to the decrease in lactic acid content during the ensilage process [30], which led to a decrease in environmental pH and inhibited the growth of *Escherichia coli*. Filamentous fungi gradually decreased with the extension of ensilage time and disappeared after 30 days of ensilage, but they were present in the CK_60 and C_30 treatment groups, which may be caused by the change in environmental components and acidity during the ensilage process [31].

### 3.2. Microbial Community in Silage

Ensiling, a fermentation process, occurs where various microorganisms interact, and the quality of silage is influenced by the composition of the bacterial communities involved in the process [5]. Fresh oat samples had a higher abundance of Proteobacteria and a lower abundance of Firmicutes. However, the lower abundance of Firmicutes significantly increased after ensiling, indicating that the fermentation process during ensiling was primarily carried out by Firmicutes utilizing carbon sources [3]. Despite the lower content of WSC and LAB in oat, the addition of exogenous fibrotic enzymes showed good results, indicating that the oat silage treated with additives obtained a higher content of metabolizable sugars and then an increased abundance of Lactobacillus [13]. Fresh oats had a higher abundance of *Pantoea* and *Enterobacter* and a very low abundance of *Lactobacillus*. However, during ensiling, *Weissella* was the dominant genus in the early stages of fermentation, but its abundance gradually decreased with increasing ensiling time. This may be due to acidification of the ensiling environment, suppression by competing genera, and a decrease in available metabolizable sugars in the raw material [30]. During the process of oat silage, there was mainly fermentation competition between the phylum Firmicutes and Proteobacteria, whose relative abundance was affected by different silage times and additives. The CK_3 treatment group showed a low abundance of Firmicutes and a high abundance of Proteobacteria, which may be due to the regulatory function of additive treatment on the microbial community composition. After 14 days of silage, the abundance of Firmicutes was higher, which may be due to the suitable fermentation conditions during the fermentation process of oat silage. The abundance of *Lactobacillus* was higher after 14, 30, and 60 days of ensiling, and both cellulase and xylanase increased the abundance of *Lactobacillus*. This may be because the time at which cellulase and xylanase act on the cell wall of oat is mainly at 14, 30, and 60 days rather than at 3 and 7 days [32]. Treatment with cellulase and xylanase increased the fermentable substrate content of oat silage, and then the abundance of *Lactobacillus* increased [33]. The abundance of *Weissella*, *Enterobacter*, and *Pediococcus* decreased notably with the treatment C_60 group; this may be the increase in plant Lactobacillus reducing the pH of the environment and inhibiting their growth [19].

Alpha diversity quantifies feature diversity within individual samples and can be compared across sample groups [34]. This was very important for evaluating the richness and evenness of bacteria during the fermentation process of silage. Exogenous fibrotic enzymes did not change the Sob, Chao, Shannon, and Simpson index in oat silage, but compared to fresh samples, the Sob, Chao, Shannon, and Simpson index increased after ensiling, which may be because the oat silage nutritional composition and the microbial population had been altered during the ensiling process [35]. The X_30, CK_60, and X_60 treatment groups had higher Alpha diversity indices, indicating an increase in bacterial species and quantity after 30 and 60 days of ensiling, possibly due to easier microbial utilization of carbon sources for growth compared to other periods [16]. Beta diversity compares the feature differences between each pair of samples and generates a distance matrix for the beta diversity distance between all samples [34]. The difference in distance matrix between different treatment groups indicates that both silage time and exogenous fibrotic enzyme treatment significantly changed the species composition and abundance during the ensiling process of oat [36], as well as the significant contribution of cellulase and xylanase.

During the fermentation process of silage, there is a strong correlation between bacteria, which promote, inhibit, or coexist with each other, ultimately affecting silage fermentation [37]. The number of nodes and edges determines the complexity of the correlation networks. This study found that the number of nodes and edges in silage for 3 days was significantly lower than 60 days. This indicated that the environment of silage for 3 days was not conducive to microbial growth, which may be due to the low WSC content of oats [19]. The C_60 treatment group showed the highest number of nodes and edges, indicating that after 60 days of ensiling, the cellulase treatment of oat provided sufficient substrate for microbial utilization. This is different from previous studies that showed more complex microbial correlation networks after 3 days of ensiling, which may be the result of different raw materials or additives [38]. However, the X_60 treatment group exhibited lower microbial correlation network complexity, which may be the result of an increase in xylan content in the environment after 60 days of ensiling, promoting the fermentation of heterofermentative LAB [14].

Predictive functional analysis is a technique that links marker gene data with available microbial genomes to predict metagenomic content and thus predict the assumed biological functions of microbial communities [39]. The metabolic levels increased gradually with the extent of ensiling time, indicating an increase in the abundance and diversity of bacteria in oat ensilage, resulting in higher metabolic levels in the environment [19]. The metabolic level under xylanase treatment is relatively low, especially under the X_30 treatment group, which may be one of the reasons for obtaining higher levels of WSC content under the X_60 treatment [40]. The C_60 treatment group showed the highest metabolic level, indicating that the glucose content in the environment was increased by cellulase treatment, promoting bacterial fermentation metabolism [41]. However, the WSC content significantly decreased after 60 days of silage, indicating that this higher metabolic level caused energy loss [38]. By analyzing the correlation between fermentation results and bacteria, it is possible to predict the bacteria that play a dominant role in the fermentation process of silage [42]. In this study, we conducted a correlation analysis between the top ten genera and species in abundance and fermentation quality and found that there were significant correlations between lactic acid and most bacteria, indicating that these bacteria were the main factors affecting oat silage fermentation [43]. There was a significant correlation between NDF and a few bacteria and no significant correlation between bacteria and ADF; this can be explained by the fact that the changes in NDF and ADF were mainly caused by exogenous fibrotic enzymes [44], but not by bacteria. Most of the bacteria were significantly positively correlated with CP, which may be one of the factors causing their content to increase. Most of the bacteria that exhibited a significant negative correlation with WSC may be one of the factors leading to its decrease in content [43]. In addition, almost all microorganisms used fermentable carbon sources for growth during the fermentation process of silage, so a decrease in WSC content after silage was inevitable [23]. In this study, the use of cellulase increased the fermentable substrate of silage, but higher bacterial abundance and metabolic levels did not preserve WSC well.

## 4. Materials and Methods

### 4.1. Silage Preparation and Treatments

The oat materials used in this study were sourced from ArKhorchin Banner (43°77′ E, 120°78′ N), Chifeng, China. After the mechanical cutting of oat during the heading period, we selected an area of 1 hectare as the experimental area. After four hours of wilting, enough samples were collected randomly for the experiment. Before the additive treatment, oat materials were cut into 2–3 cm using a forage chopper and thoroughly mixed. Prior to ensiling, the oat had a dry matter content of 289 g/kg DM. We have set up 2 additive treatment combinations for 5 silage time periods: (1) control (CK, distributed water); (2) silages inoculated with xylanase (X); and (3) silages inoculated with cellulase (C). Two exogenous fibrotic enzymes are provided by Shanghai Macklin Blochemical Co. Ltd., (Shanghai, China); silage time periods were 3, 7, 14, 30, and 60 days, respectively. Xylanase and cellulase activities were 100,000 μ/g and 10,000 μ/g, respectively, and they were all asepsis. The concentrations of cellulase and xylanase added in this study were both 1000 μ/kg FW. The cellulase and the xylanase were diluted with distilled water so that they were applied at the same rate (10,000 μ/g FM); equal amounts of sterile distilled water were sprayed onto untreated forage. The oat forage in the given amount was sprayed with diluted additives (one sprayer for each treatment). Approximately 300 g of grass forage were vacuum-sealed in polyethylene bags (25 × 35 cm). Each processing group was repeated four times. All silage samples were stored at room temperature (25 °C ± 2) with no light. Samples for analysis were opened after ensiling for 3, 7, 14, 30, and 60 days. These samples were taken randomly for chemical composition, fermentation quality, microbial count, and microbial composition analysis.

### 4.2. Chemical Composition, Fermentation Composition, and Microorganisms-Colony Count

Oat silage samples were dried at 65 °C for 48 h using an air dryer to determine the dry matter (DM) content in the sample [19]. Then, all dried oat silage samples were crushed by a cutting grinder (SM200) to obtain 40 mesh samples for chemical composition analysis. Analysis of WSC content in samples using anthrone colorimetry [45]. The full-automatic Kjeltec (Model: 8400, FOSS, Hilleroed, Denmark) was used to measure the CP of the samples [46]. The ANKOM fiber analyzer (Model: A2000i, Beijing Anke Borui Technology Co., Ltd., Beijing, China) was used to determine the ADF and NDF of the silage samples by the method of Van Soest et al. [47]. The muffle furnace (Model: SX2-10-12N, Beijing Anke Borui Technology Co., Ltd., Beijing, China) has been employed to determine the crude ash content of the samples [48].

To obtain the liquid extract, a sterile homogenizer was utilized in this study with a sample-to-sterile water ratio of 10:90. After tapping and thorough mixing, the liquid was filtered through four layers of coarse cotton cloth and filter paper. The pH value was immediately determined using a pH meter (Model: LEICI pH S-3C, Shanghai Yitian Scientific Instrument Co., Ltd., Shanghai, China), and then the liquid was frozen in an ultra-low temperature refrigerator at −80 °C to prepare for the subsequent determination of organic acids. The organic acid content of the samples was analyzed using high-performance liquid chromatography (Model: Waters E2695, Milford, MA, USA). The content of ammonia nitrogen (NH_3_-N) was determined using the phenol-hypochlorous acid colorimetric method [49].

### 4.3. Microbial Sequencing and Statistical Analysis

All samples for microbial DNA analysis were carried out using experimental methods such as Wang et al. [19], and the online analysis platform Omicromart (http://www.omicsmart.com, accessed on 5 November 2023) was used for the analysis of bacterial sequencing data, including species abundance composition, alpha diversity, beta diversity, and others. Chemical composition and organic acid data were carried out using IBM SPSS Statistics 26 software and GraphPad Prism 8 to draw charts. Perform a two-way analysis of variance with additives and fermentation time as the main variables. Using Duncan multiple comparisons to determine statistical differences between mean values and considering them to be significant at *p* < 0.05.

## 5. Conclusions

There was a better-expected result from cellulase and xylanase treatments to assist in the fermentation of oat silage. The exogenous fibrotic enzyme reduced the NDF, ADF, cellulose, and hemicellulose content in oat silage, resulting in an increased concentration of metabolizable sugars in the environment, and the X_60 treatment group has a significant residual WSC content. The abundance of *Lactobacillus* significantly increased in the X_30, C_30, X_60, and C_60 treatment groups, contributing to higher silage quality during fermentation. The treatment of cellulase increased the microbial abundance of oat silage and created more complex correlation networks. In short, although the oat material has low LAB attachment and WSC content, when treated with exogenous fibrotic enzymes, higher fermentation and nutritional quality were obtained; however, the high metabolic level caused by cellulase treatment may also lead to energy loss during the ensiling process.

## Data Availability

The sequencing data for the 16S rRNA gene sequence were stored at NCBI with BioProject accession number PRJNA1019013.

## Supplementary Materials

The following supporting information can be downloaded at: https://www.mdpi.com/article/10.3390/plants13010006/s1, Table S1: Chemical composition of oat before ensiling; Table S2: Nutritional quality of oat silage during different treatment and period of ensiling; Table S3: Fermentation quality of oat silage during different treatment and period of ensiling.
