# Supplementary material for: Study on Dynamic Fermentation of Oat Silage Assisted by Exogenous Fibrolytic Enzymes
Source: Plants (Basel). 2023 Dec 19;13(1):6. doi: 10.3390/plants13010006 (PMC10780392; doi:10.3390/plants13010006)
Supplement: Supplementary file 1 [file plants-13-00006-s001.zip › plants-2692228-supplementary.pdf]

plants

Supplementary Material

Table S1. Chemical composition of oat before ensiling.

Items	Oat silage	SEM
pH	6.41	0.014
DM (g/kg FW)	290.24	0.218
NAF (g/kg DM)	663.04	0.455
ADF (g/kg DM)	378.19	0.325
ADL (g/kg DM)	56.80	0.149
CP (g/kg DM)	123.47	0.068
Cellulose (g/kg DM)	321.38	0.035
Hemicellulose (g/kg DM)	284.85	0.089
WSC (g/kg DM)	62.13	0.087
Ash (g/kg DM)	62.19	0.056
Lactic acid bacteria (lg CFU/g)	1.51	0.006
Yeast (lg CFU/g)	2.01	0.038
Filamentous fungi (lg CFU/g)	5.08	0.014

Table S2. Nutritional quality of oat silage during different treatment and period of ensiling.

Items[1]	Treatment[2]	Ensilage time[3]					SEM[4]	Significance[5]		
		3	7	14	30	60		T	D	T×D
DM (g/kg FW)	CK	296.97^{Ab}	297.27^{Ab}	308.47^{Aa}	298.83^{Ab}	307.70^{Aa}	0.064	**	*	ns
	X	287.00^{Bb}	278.60^{Bc}	283.60^{Bb}	282.23^{Bb}	297.20^{Ba}	0.022			
	C	294.97^{Aa}	280.50^{Bb}	281.47^{Bb}	280.90^{Bb}	294.03^{Ba}	0.068			
NDF (g/kg DM)	CK	638.54^{Aa}	639.07^{Aa}	627.99^{Ab}	619.72^{Ac}	607.34^{Ad}	0.005	**	**	ns
	X	626.70^{Ba}	589.41^{Bc}	603.19^{Bb}	597.36^{Bb}	568.63^{Bd}	0.005			
	C	602.49^{Ca}	582.74^{Bb}	596.39^{Ba}	571.25^{Cc}	550.77^{Cd}	0.014			
ADF (g/kg DM)	CK	329.43^{Cc}	348.47^{a}	352.40^{Aa}	350.80^{Aa}	346.57^{Ab}	0.118	**	**	**
	X	344.90^{Aa}	341.03^{a}	338.30^{Bb}	315.50^{Bc}	321.33^{Bc}	0.065			
	C	339.33^{Ba}	344.07^{a}	322.33^{Cb}	320.23^{Bb}	308.23^{Cc}	0.085			
ADL (g/kg DM)	CK	46.53	39.48^{B}	48.70	46.38	42.95^{B}	0.007	ns	ns	ns
	X	50.46	52.58^{A}	47.79	44.49	45.72^{B}	0.004			
	C	51.64	49.63^{A}	46.85	47.64	50.66^{A}	0.003			
Hemicellulose (g/kg DM)	CK	309.10^{Aa}	290.59^{Ab}	275.59^{Ac}	250.95^{Be}	260.76^{Ad}	0.014	**	**	**
	X	281.80^{Ba}	248.36^{Bc}	264.86^{Bb}	268.52^{Ab}	247.28^{Bc}	0.019			
	C	263.18^{Cb}	238.66^{Cc}	274.03^{Aa}	242.68^{Bc}	242.56^{Bc}	0.018			
Cellulose (g/kg DM)	CK	282.91^{Bb}	309.00^{Aa}	303.70^{Aa}	304.39^{Aa}	303.63^{Aa}	0.014	**	*	**
	X	294.44^{Aa}	288.46^{Bb}	290.54^{Ba}	271.01^{Bc}	275.63^{Bc}	0.008			
	C	287.67^{Ba}	294.46^{Ba}	275.50^{Cb}	272.59^{Bb}	257.56^{Cc}	0.009			
CP (g/kg DM)	CK	133.85^{c}	140.29^{Aa}	137.28^{Ab}	138.74^{b}	142.94^{a}	0.002			
	X	134.82^{c}	134.56^{Bc}	136.88^{Ab}	137.07^{b}	144.60^{a}	0.002	ns	**	**
	C	134.31^{c}	139.66^{Ab}	131.25^{Bc}	138.02^{b}	142.65^{a}	0.002			
WSC (g/kg DM)	CK	31.52^{Ba}	24.78^{b}	21.53^{Bb}	19.78^{c}	17.89^{Bc}	0.001	ns	**	**
	X	30.80^{Ba}	23.32^{b}	23.48^{Ab}	20.53^{b}	21.90^{Ab}	0.002			
	C	33.08Aa	24.37^{b}	25.97^{Ab}	21.94^{c}	16.58^{Bd}	0.001			

The values with different lowercase letters show significant differences among ensilage time in the same additive treatment, the values with different capital letters show significant differences among additives in the same ensilage time(days) ($P < 0.05$), ns, not significant; *$P < 0.05$; **$P < 0.01$.

1. FW, fresh weight; DM, dry matter; NDF, neutral detergent fiber; ADF, acid detergent fiber; ADL, acid detergent lignin; CP, crude protein; WSC, water-soluble carbohydrates; SEM, standard error of the mean.
2. CK, control, no additive; X, silages inoculated with xylanase; C, silages inoculated with cellulase;.
3. 3, 7, 14, 30 and 60 were different ensilage time (days).
4. SEM, standard error of means.

5. T, additives treatment; D, ensilage time (days); T × D, the interaction between additives and days.

Table S3. Fermentation quality of oat silage during different treatment and period of ensiling.

Items	Treatment[1]	Ensilage time[2]					SEM[3]	Significance[4]		
		3	7	14	30	60		T	D	T×D
pH	CK	4.97^{b}	5.48^{Aa}	5.46^{Aa}	4.98^{Ab}	4.63^{Ac}	0.175	**	**	ns
	X	4.85^{b}	5.43^{Aa}	5.03^{Bb}	4.77^{Bb}	4.46^{Bc}	0.154			
	C	4.94^{a}	5.05^{Ba}	4.85^{Cb}	4.68^{Bb}	4.39^{Bc}	0.078			
Lactic acid (g/kg DM)	CK	22.64^{Bd}	25.21^{Bd}	32.86^{Bc}	41.64^{Bb}	60.18^{Ba}	1.609	**	**	**
	X	24.18^{Ad}	22.82^{Cd}	37.91^{Ac}	51.36^{Ab}	71.92^{Aa}	1.555			
	C	25.16^{Ad}	27.61^{Ad}	40.47^{Ac}	56.30^{Ab}	75.18^{Aa}	1.215			
Acetic acid (g/kg DM)	CK	7.02^{Bc}	8.18^{Bc}	13.16^{Bb}	21.02^{Ca}	24.36^{Ba}	0.683	**	**	**
	X	8.80^{Ac}	10.66^{Ac}	15.99^{Ab}	26.70^{Ba}	31.90^{Aa}	0.532			
	C	9.06^{Ac}	11.68^{Ac}	15.89^{Ab}	30.25^{Aa}	35.98^{Aa}	0.554			
Butyric acid (g/kg DM)	CK	2.93^{A}	3.06^{A}	3.18^{A}	3.27^{A}	3.19^{A}	0.539	**	ns	ns
	X	2.61^{B}	2.71^{B}	2.84^{B}	2.62^{B}	2.52^{B}	0.076			
	C	2.49^{C}	2.61^{B}	2.71^{B}	2.67^{B}	2.49^{B}	0.154			
Ammoniacal nitrogen (g/kg DM)	CK	23.23^{Aa}	19.27^{Cb}	20.22^{Ab}	21.78^{Ab}	17.45^{Bc}	0.145	**	**	**
	X	21.70^{Ba}	20.26^{Ba}	18.40^{Bb}	17.65^{Bb}	15.25^{Cc}	0.014			
	C	19.04^{Ca}	21.30^{Aa}	18.33^{Bb}	18.31^{Bb}	19.42^{Aa}	0.035			
Lactic acid bacteria (lg CFU/g)	CK	5.46^{Bc}	6.71^{Ba}	6.25^{Bb}	6.98^{Aa}	6.58^{Ba}	0.234	*	**	ns
	X	5.99^{Ac}	7.11^{Aa}	6.70^{Aa}	6.48^{Bb}	6.86^{Aa}	0.322			
	C	5.51^{Bc}	6.60^{Ba}	6.59^{Aa}	6.26^{Bb}	6.73^{ABa}	0.229			
Yeasts (lg CFU/g)	CK	4.78^{Bb}	6.31^{Ba}	6.35^{Aa}	6.58^{a}	6.35^{a}	0.589	ns	ns	*
	X	6.44^{Ab}	7.06^{Aa}	5.84^{Bc}	6.70^{a}	6.47^{b}	0.307			
	C	6.24^{A}	6.03^{B}	6.58^{A}	6.45	6.33	0.317			
Escherichia coli (lg CFU/g)	CK	5.09^{Ba}	3.43^{Bb}	ND	ND	ND	1.913	ns	**	ns
	X	5.35^{Ba}	3.69^{Bb}	ND	ND	ND	1.510			
	C	6.97^{Aa}	5.31^{Ab}	ND	ND	ND	0.936			
Filamentous fungi (lg CFU/g)	CK	6.66^{Ba}	5.45^{Bb}	6.15^{Ba}	ND	3.35^{Ac}	0.753	**	**	**
	X	6.34^{Ca}	5.82^{Ab}	5.79^{Cb}	ND	ND	0.315			
	C	7.34^{Aa}	5.82^{Ac}	6.62^{Ab}	ND	1.03^{Bd}	0.561			

The values with different lowercase letters show significant differences among ensilage time in the same additive treatment, the values with different capital letters show significant differences among additives in the same ensilage time(days) (P<0.05), ns, not significant; *P < 0.05; **P < 0.01. ND; not detected.

1. CK, control, no additive; X, silages inoculated with xylanase; C, silages inoculated with cellulase;
2. 3, 7, 14, 30 and 60 were different ensilage time (days).
3. SEM, standard error of means.
4. T, additives treatment; D, ensilage time (days); T × D, the interaction between additives and days.
